# Association between Pre-Pregnancy Overweightness/Obesity and Pregnancy Outcomes in Women with Polycystic Ovary Syndrome: A Systematic Review and Meta-Analysis

**DOI:** 10.3390/ijerph19159094

**Published:** 2022-07-26

**Authors:** Szu-Ting Yang, Chia-Hao Liu, Sheng-Hsiang Ma, Wen-Hsun Chang, Yi-Jen Chen, Wen-Ling Lee, Peng-Hui Wang

**Affiliations:** 1 Department of Obstetrics and Gynecology, Taipei Veterans General Hospital, Taipei 112, Taiwan; styang6@vghtpe.gov.tw (S.-T.Y.); mikeliuu@gmail.com (C.-H.L.); chenyj@vghtpe.gov.tw (Y.-J.C.); 2 Faculty of Medicine, School of Medicine, National Yang Ming Chiao Tung University, Taipei 112, Taiwan; thetinynotes@gmail.com; 3 Institute of Clinical Medicine, National Yang Ming Chiao Tung University, Taipei 112, Taiwan; johnweiwang@gmail.com; 4 Department of Dermatology, Taipei Veterans General Hospital, Taipei 112, Taiwan; 5 Department of Nursing, Taipei Veterans General Hospital, Taipei 112, Taiwan; 6 Department of Medicine, Cheng-Hsin General Hospital, Taipei 112, Taiwan; 7 Department of Medical Research, China Medical University Hospital, Taichung 404, Taiwan; 8 Female Cancer Foundation, Taipei 104, Taiwan

**Keywords:** polycystic ovary syndrome, pregnancy outcomes, pre-pregnancy overweightness, pre-pregnancy obesity

## Abstract

Polycystic ovary syndrome (PCOS) is a common metabolic problem in women of reproductive age. Evidence suggests pregnant women with PCOS may have a higher risk of the development of adverse pregnancy outcomes; however, the relationship between pre-pregnancy overweight/obesity and pregnancy outcomes in women with PCOS remains uncertain. We try to clarify the relationship between pre-pregnancy overweight/obesity and subsequent pregnancy outcomes. Therefore, we conducted this systematic review and meta-analysis. We used the databases obtained from the PubMed, Embase, Web of Science, and Cochrane databases, plus hand-searching, to examine the association between pre-pregnancy overweightness/obesity and pregnancy outcomes in women with PCOS from inception to 4 February 2022. A total of 16 cohort studies, including 14 retrospective cohort studies (*n* = 10,496) and another two prospective cohort studies (*n* = 818), contributed to a total of 11,314 women for analysis. The meta-analysis showed significantly increased odds of miscarriage rate in PCOS women whose pre-pregnancy body mass index (BMI) is above overweight (OR 1.71 [95% CI 1.38–2.11]) or obese (OR 2.00 [95% CI 1.38–2.90]) under a random effect model. The tests for subgroup difference indicated the increased risk was consistent, regardless which body mass index cut-off for overweight (24 or 25 kg/m^2^) or obesity (28 and 30 kg/m^2^) was used. With the same strategies, we found that pregnant women in the control group significantly increased live birth rate compared with those pregnant women with PCOS as well as pre-pregnancy overweight/obesity (OR 0.79 [95% CI 0.71–0.89], OR 0.78 [95% CI 0.67–0.91]). By contrast, we did not find any association between PCOS women with pre-pregnancy overweight/obesity and preterm birth. Based on the aforementioned findings, the main critical factor contributing to a worse pregnancy outcome may be an early fetal loss in these PCOS women with pre-pregnancy overweight/obesity. Since PCOS women with pre-pregnancy overweightness/obesity were associated with worse pregnancy outcomes, we supposed that weight reduction before attempting pregnancy in the PCOS women with pre-pregnancy overweightness/obesity may improve the subsequent pregnancy outcomes.

## 1. Introduction

Polycystic ovary syndrome (PCOS) affects 4–18% of women of reproductive age, which is characterized by hyperandrogenism, chronic anovulation, and a picture of multiple small follicular cysts in the ovary [1,2,3,4,5]. PCOS is closely linked to metabolic and reproductive disorders, such as obesity, insulin resistance (IR), anovulation-related infertility and a thickened endometrium [6,7]. Furthermore, women with PCOS were associated with greater risk of obstetric complications, including preterm birth (PB), miscarriage, perinatal death, preeclampsia, gestational diabetes mellitus (GDM), and cesarean sections (C/S) [8].

Around 80% of women with PCOS have a higher body mass index (BMI) value, reaching overweightness or obesity [9]. Previous studies have explored the relationship between higher BMI and adverse pregnancy outcomes, including GDM, preeclampsia, intrauterine death, and C/S in general population [10]. However, whether higher pre-pregnancy BMI affects the outcome of pregnancy in women with PCOS is unclear [11]. Taking miscarriage as an example, some studies found that the miscarriage rate was similar among different BMI categories [12,13,14,15,16]. Nevertheless, some studies suggested a higher miscarriage rate in overweight or obese women with PCOS [17,18,19,20]. Conversely, one study reported that miscarriage rate was higher in lean women with PCOS, although the difference did not reach significance [21]. The association between pre-pregnancy BMI value and pregnancy outcomes remains inconclusive in women with PCOS. Additionally, we believe the fact that worse pregnancy outcomes are present in pregestational overweight/obese women, regardless of whether they are diagnosed with PCOS or not [22,23,24,25,26]. Therefore, we thus performed this systematic review and meta-analysis to elucidate the association between pre-pregnancy overweightness/obesity and pregnancy outcomes in the PCOS women. The results may improve personalized risk assessment in PCOS women and can be useful for the guidance of further therapeutic plan and policy to provide a better chance to enhance pregnancy outcomes in PCOS women with pre-pregnancy overweightness/obesity who plan the pregnancy.

## 2. Materials and Methods

### 2.1. Search Strategy

This meta-analysis was registered in PROSPERO (CRD42022300037) and was conducted adhering to the Meta-analyses of Observational Studies in Epidemiology (MOOSE) checklist and the Preferred Reporting Items for Systematic Reviews and Meta-Analyses (PRISMA) guidelines [27,28,29,30]. Two reviewers (Drs. Yang and Liu) independently performed the literature search, data extraction, and quality assessment. Any disagreement was resolved by consensus among the reviewers or referred to a third reviewer (Dr. Wang).

The PubMed, Embase, Web of Science, and Cochrane databases, plus hand-searching, were searched for relevant studies from the respective inception of these databases to 4 February 2022. The Medical Subject Headings (MeSH) and Emtree were identified using the selected search terms and were combined using appropriate Boolean operators (Table S1). Both reviewers independently selected relevant studies by scanning the titles and abstracts of search results. The full text of potential studies was obtained and examined for eligibility.

### 2.2. Inclusion and Exclusion Criteria

The key questions for the systematic literature review were based on the Populations, Intervention, Comparison, Outcome, and Study Design (PICOSD) framework as follows: (1) The study population was pregnant women diagnosed with PCOS; (2) The case group consists of patients with pregestational overweightness or obesity. The control group consists of women without overweightness or obesity; and (3) The outcomes of interest for this review included miscarriage, fetal death, PB, pregnancy-induced hypertension, pre-eclampsia, GDM, C/S, fetal macrosomia, and intrauterine growth restriction (IUGR). Case reports, case series, reviews, comments, letters, and conference abstracts were excluded. Only articles published in English and conducted on humans were included.

### 2.3. Data Extraction and Risk of Bias Assessment

The following data were extracted from the included studies: first author, year of publication, country, study design, and characteristics of participants (sample size, mean age, mean BMI), conception method, causes of infertility, diagnostic criteria for PCOS, the outcome variables and the definition of outcomes. The quality of the selected studies was assessed using the Newcastle-Ottawa quality assessment scale with a maximum score of 9 representing the highest quality [31,32].

### 2.4. Statistical Analysis

The statistical analyzes were performed using RevMan software version 5.4.1 (The Cochrane Collaboration, 2020.) and Comprehensive Meta-Analysis Version 3.3.070 (20 November 2014). Odds ratios (ORs) were calculated for dichotomous outcomes with a 95% confidence interval (Cl). A random-effects model was used for analysis because of possible different characteristics existing between the studies. The inconsistency test (I^2^) was used to assess statistical heterogeneity across the included studies. Publication bias was assessed using Egger’s regression test with 2-tailed *p*-value.

## 3. Results

### 3.1. Study Selection

**Figure 1:**
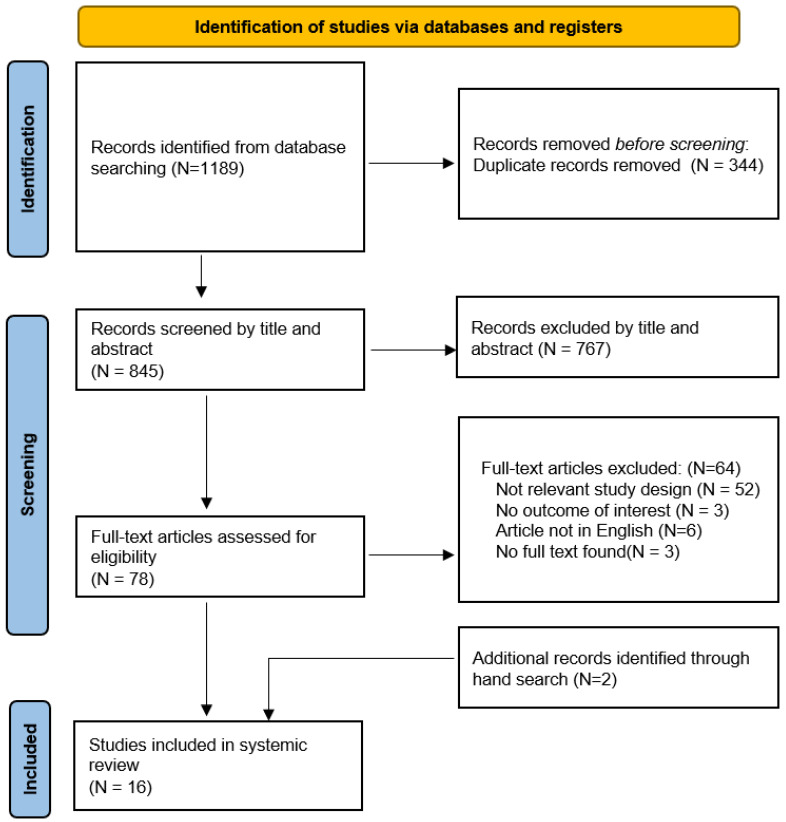
Flowchart of literature search and selection.

As shown in Figure 1, a total of 1189 studies were identified by our search. A total of 344 studies were removed due to duplicate records, and 767 studies were excluded after assessing the title or abstract. A total of 78 studies remained for full-text review and 64 studies were not eligible for criteria, including 52 not relevant study design, 3 without outcome of interest, 6 where the article is not in English, and 3 studies without full text found. Two other articles were identified after searching the reference lists. A total of 16 studies fulfilled the eligibility criteria.

### 3.2. Study Characteristics

Table 1 summarizes the characteristics of the 16 studies which contain a total of 11,314 women. Fourteen of the 16 studies were retrospective cohort studies (*n* = 10,496) and another 2 studies were prospective cohort studies (*n* = 818). The majority of the studies were conducted in China (10 studies, *n* = 10,737). Of the 16 studies, the Newcastle-Ottawa Scale score ranged from 6 to 9 (median 7).

The classifications of underweight, normal weight, overweight, and obese were not consistent among these studies. World Health Organization (WHO) classification for nutritional status is defined by underweight as <18.5 kg/m^2^, normal weight as 18.5–24.9 kg/m^2^, overweight as 25.0–29.9 kg/m², and obese as BMI ≥ 30 kg/m^2^ [2,33], and most of studies adopted this classification [13,15,16,17,18,19,34,35,36]. However, some studies used 24 kg/m^2^ and 28 kg/m^2^ as cut-off points for overweightness and obesity [37], respectively, especially studies conducted in China [12,14,20,21,38,39]. Regarding the criteria of PCOS, 15 studies used the 2003 Rotterdam criteria to diagnose PCOS, while only one study in 1992 used ultrasound images as diagnostic criteria [40].

**Table 1:** Characteristics of the included studies.

Study [Reference], Year (Country)	Study Design	Study Population			Outcome			PCOS Criteria	Con Method	Detail of Con Method	NOS Score
		BMI (kg/m^2^)	Sample Size	Age(y)	Miscarriage A: Definition of Miscarriage B: Definition of Miscarriage (M) Rate	Live Birth A: Definition of Live Birth B: Definition of Live Birth (L) Rate	Preterm Birth A: Definition of Preterm Birth B: Definition of Preterm Birth (P) Rate				
Hamilton-Fairley et al. [40]. 1992 (UK)	RC	≧25.0, <28	25	30.9 ± 3.7	A: not mentioned B: M/positive pregnancy test			Adams (US)	OI	Gonadotrophin	9
		≧19, <25	75	29.8 ± 4.3							
McCormick et al. [15]. 2008 (USA)	RC	≧30	10	31.5 ± 5.0	A: GA < 20 weeks B: M/positive pregnancy test	A: alive newborn B: L/egg retrievals		Rot	IVF	GnRH agonist protocol (97.9%); GnRH-anta protocol (2.1%)	9
		≧18.5, <30	6	31.5 ± 3.0							
Ozgun et al. [19]. 2011 (Turkey)	PC	≧30	18	26.7 ± 2.9	A: GA < 20 weeks B: M/clinical pregnancy	A: not mentioned B: L/all patients		Rot	ICSI	Long protocol	8
		<30	26	26.8 ± 4.5							
Shalom-Paz et al. [13]. 2011 (Canada)	RC	≧35,	13	29.6 ± 1.0	A: not mentioned B: M/clinical pregnancy	A: not mentioned B: L/clinical pregnancy		Rot	IVF	Not mentioned	7
		≧30, <35	12	31.0 ± 0.9							
		≧25, <30	24	31.2 ± 0.7							
		≧20, <25	50	30.8 ± 0.4							
		<20	17	31.3 ± 0.8							
De Frène et al. [34]. 2014 (Belgium)	RC	>25	93	29.0 ± 4.2	A: GA < 25 weeks B: M/positive hCG at GA 4 weeks		A: GA < 37 weeks B: P/live birth	Rot	Any	Not mentioned	8
		≦25	107	28.4 ± 3.1							
Huang et al. [21]. 2014 (China)	RC	≧24	49	30.5 ± 4.1	A: not mentioned B: M/clinical pregnancy	A: alive and survived > 1 month B: L/all patients		Rot	IVF/ICSI	Not mentioned	7
		<24	79	29.4 ± 3.4							
Bailey et al. [35]. 2014 (USA)	RC	≧30	31	32.4 ± 3.2	A: GA < 20 weeks B: M/FET cycles	A: alive newborn B: L/FET cycles		Rot	IVF/ICSI	Not mentioned	8
		≧25, <30	19	32.6 ± 2.9							
		≧18.7, <25	51	32.0 ± 3.5							
Cui et al. [20]. 2016 (China)	RC	≧28	88	27.50 ± 3.4	A: GA < 20 weeks B: M/clinical pregnancy		A: not mentioned B: P/clinical pregnancy	Rot	IVF/ICSI	Long protocol	6
		≧24, <28	125	27.50 ± 3.1							
		≧18.5, <24	183	26.99 ± 2.9							
		<18.5	12	27.33 ± 3.3							
Sheng et al. [12]. 2017 (China)	PC	≧28	63	28.7 ± 2.7	A: <1 st trimester B: M/clinical pregnancy			Rot	IVF/ICSI	Long protocol	7
		≧24, <28	211	28.5 ± 3.1							
		≧18.5, <24	449	27.7 ± 3.1							
		<18.5	51	26.3 ± 3.1							
Pan et al. [14]. 2018 (China)	RC	≧28	102	29.77 ± 3.5	A: GA < 28 weeks B: M/ET	A: alive newborn B: L/ET	A: GA 28~37 weeks B: P/ET	Rot	IVF	Long protocol	6
		≧24, <28	315	29.45 ± 3.5							
		≧18.5, <24	606	29.08 ± 3.2							
		<18.5	51	27.78 ± 3.1							
Yang et al. [36]. 2018 (China)	RC	≥25	213	29.5	A: not mentioned B: M/clinical pregnancy	A: not mentioned B: not mentioned	A: not mentioned B: P/clinical pregnancy	Rot	IVF	GnRH-anta protocol	7
		<25	370	29.1							
Chen et al. [39]. 2018 (China)	RC	≥24	138	28.9 ± 3.0	A: GA < 12 weeks B: M/clinical pregnancy	A: not mentioned B: L/all patients	A: not mentioned B: P/live birth	Rot	IVF	GnRH-anta protocol	7
		<24	260	28.8 ± 2.7							
Lin et al. [16]. 2019 (China)	RC	≧30	228	33.27 ± 3.6	A: not mentioned B: M/clinical pregnancy	A: alive at GA ≥ 24 weeks B: L/FET cycles	A: <37 weeks B: P/FET cycles	Rot	IVF/ICSI	GnRH-anta protocol; mild stimulation; PPOS (Percentage not mentioned)	7
		≧25.0, <30	480	33.09 ± 3.8							
		≧18.5, <25	972	32.82 ± 3.4							
Qiu et al. [18]. 2019 (China)	RC	≧30	204	30.05 ± 3.6	A: <1 st trimester B: M/clinical pregnancy	A: alive at GA ≥24 weeks B: L/FET cycles		Rot	IVF/ICSI with freeze all	GnRH-anta protocol	6
		≧25, <30	780	30.48 ± 3.9							
		≧18.5, <25	1911	29.97 ± 3.3							
		<18.5	184	28.91 ± 3.2							
Zhou et al. [17]. 2020 (China)	RC	≧30	198	27.97 ± 3.0	A: not mentioned B: M/clinical pregnancy	A: not mentioned B: not mentioned	A: not mentioned B: not mentioned	Rot	IVF/ICSI	Ultra-long protocol	7
		≧25, <30	742	28.19 ± 3.1							
		≧18.5, <25	800	28.13 ± 3.1							
		<18.5	42	27.76 ± 2.5							
Guan et al. [38]. 2021 (China)	RC	≧28	194	not mentioned	A: not mentioned B: M/clinical pregnancy	A: not mentioned B: L/clinical pregnancy		Rot	OI with IUI	CC (3.9%); LE (18.1%); hMG (11.3%); hMG + CC(17.8%); hMG + LE (48.9%)	7
		≧24, <28	321								
		≧18.5, <24	299								
		<18.5	17								

RC, retrospective cohort; PC, prospective cohort; GA, gestational age; Rot, Rotterdam; US, ultrasound; Con, conception, IVF, in vitro fertilization; ICSI, intracytoplasmic sperm injection; OI, ovulation induction; IUI, intrauterine insemination; ET, embryo transfer; FET, frozen embryo transfer; Any, Spontaneous pregnancy, timed-coitus, IUI, IVF/ICSI; GnRH-anta, gonadotropin releasing hormone antagonist; PPOS, progestin-primed ovarian stimulation; CC, clomiphene; LE, letrozole; hMG, human menopausal gonadotropin.

The conception method in most of the studies were in vitro fertilization (IVF) or intracytoplasmic sperm injection (ICSI) (13 studies). Ovulation induction was used in one study [40], ovulation induction with intrauterine insemination (IUI) in one study [38], and any conception method (including spontaneous pregnancy, timed-coitus, IUI, IVF/ICSI) in one study [34]. Among IVF or ICSI studies, four studies used long protocol [12,14,19,20], one study applied ultra-long protocol [17], three studies were conducted with GnRH antagonist protocol [18,36,39], two studies used various protocols [15,16], and three studies did not mention which protocol they used [13,21,35]. Regarding the causes of infertility, five studies excluded tubal factor [15,17,36,38,40], five studies excluded male factor [14,21,36,38,40], and three studies exclude oocyte cryopreservation or donation [13,14,21]. The detail information of infertility cause was presented in Table S2.

As only few studies reported outcomes of pregnancy-induced hypertension, pre-eclampsia, GDM, fetal death, C/S, fetal macrosomia, and IUGR, we focused our analysis on miscarriage, live birth, and PB after summarizing the outcomes of studies. Various definitions of miscarriage, live birth, and PB were adapted in these studies. Miscarriage was defined before first trimester in three studies [12,18,39], at gestational age before 20 weeks in four studies [15,19,20,35], at gestational age before 25 weeks in one study [34], at gestational age before 28 weeks in one study [14], and not mentioned in the remaining seven studies [13,16,17,21,36,38,40]. Miscarriage rate was defined as the number of miscarriages among the number of clinical pregnancies in most of the studies [12,13,16,17,18,19,20,21,36,38,39]. The definition of live birth was alive newborn in three studies [14,15,35], alive newborn at gestational age ≥24 weeks in two studies [16,18], alive newborn surviving more than one month after birth in one study [21], and not mentioned in the rest of the studies [13,17,19,36,38,39]. Live birth rate was defined differently, including live birth among number of all patients in three studies [19,21,39], the number of clinical pregnancies in two studies [13,38], the number of embryos transferred (ET) in one study [14], and the number of frozen embryo transfer (FET) cycles in three studies [16,18,35]. As for PB, there were two studies defining PB as delivery before 37 weeks [16,26], one study defining PB as delivery between 28 to 37 weeks [14], and three study not mentioning [20,36,39].

### 3.3. Miscarriage

**Figure 2:**
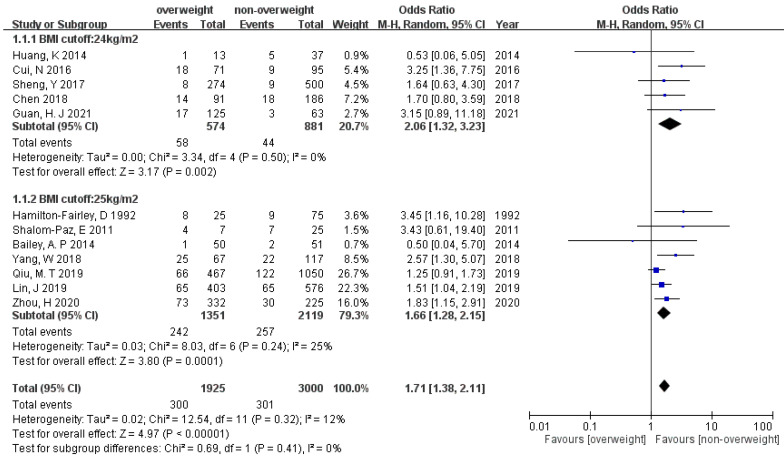
Forest plot of the association between miscarriage and overweightness in women with PCOS.

**Figure 3:**
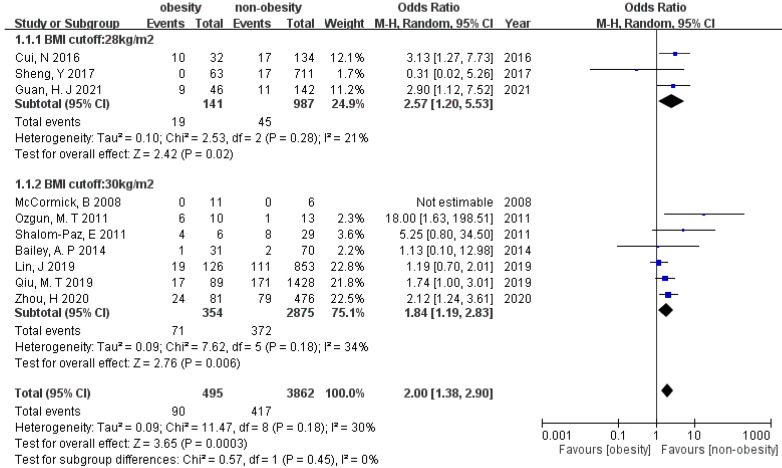
Forest plot of the association between miscarriage and obesity in women with PCOS.

All the sixteen studies provided information on miscarriage. We excluded studies conducted by De Frène et al. defining miscarriage up to 25 weeks of gestation and Pan et al. defining miscarriage up to 28 weeks of gestation, because miscarriage is generally defined as a nonviable fetus up to 20 or 24 weeks of gestation. Twelve studies analyzed the association between miscarriage and overweightness (Figure 2). The meta-analysis showed significantly increased odds of miscarriage in PCOS women whose pre-pregnancy BMI is above overweight (OR 1.71 [95% CI 1.38–2.11]) under a random effect model. Heterogeneity (I^2^) was 12%. Egger’s test showed no significant publication bias (*p* = 0.23). Subgroup analysis of BMI cut-off value with 24 or 25 kg/m^2^ was performed, showing consistent results in these two subgroups (OR 2.06 [95% CI 1.32–3.23], and OR 1.66 [95% CI 1.28–2.15], *p* = 0.41). In regard to the association between miscarriage and obesity, the result showed increased odds of miscarriage in the obese group (OR 2.00 [95% CI 1.38–2.90]) with low heterogeneity (I^2^ = 30%) (Figure 3). Egger’s test resulted no publication bias (*p* = 0.38). Moreover, subgroup analysis did not show difference between BMI cut-off 28 and 30 kg/m^2^ (OR 2.57 [95% CI 1.20–5.53], and OR 1.84 [95% CI 1.19–2.83], *p* = 0.45).

### 3.4. Live Birth

**Figure 4:**
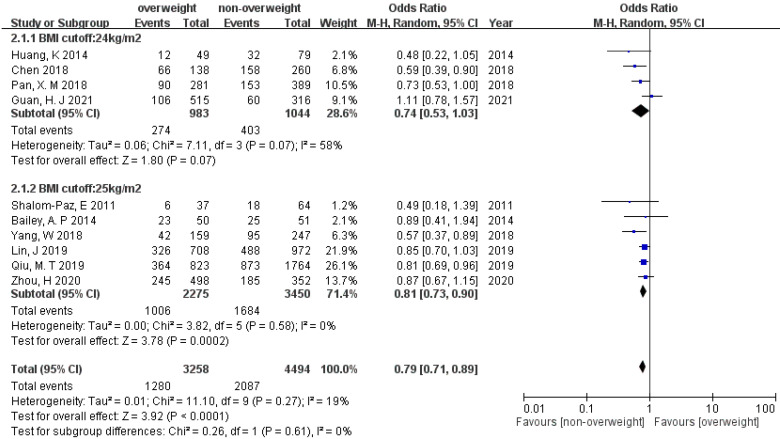
Forest plot of the association between live birth and overweightness in women with PCOS.

**Figure 5:**
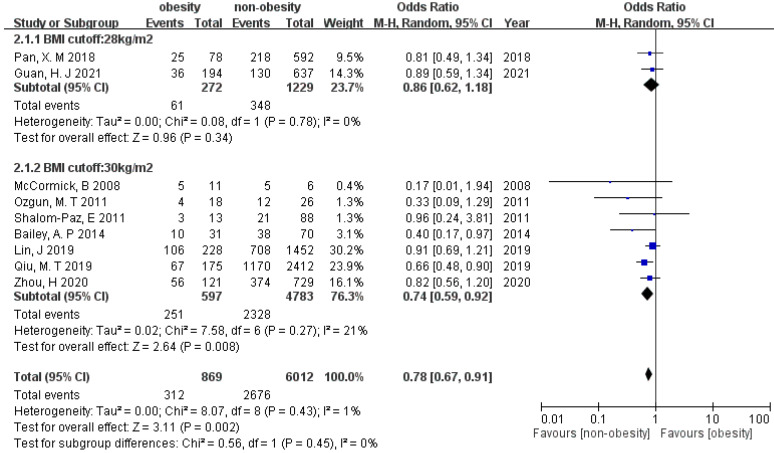
Forest plot of the association between live birth and obesity in women with PCOS.

Twelve studies included data on live birth. Using a random-effects model, the result suggested that patients in the non-overweight/non-obese groups significantly increased live birth rate compared with patients in overweight/obese groups (OR 0.79 [95% CI 0.71–0.89], and OR 0.78 [95% CI 0.67–0.91]) with low heterogeneity (19% and 1%) (Figure 4 and Figure 5). Egger’s test suggested non-significant publication bias (*p* = 0.17 and 0.08). The tests for subgroup difference indicated that there is no statistically significant subgroup effect between BMI cut-off 24 and 25 kg/m^2^, as well as between 28 and 30 kg/m^2^ (*p* = 0.61 and 0.45, respectively), suggesting different BMI cut-off does not modify the effect of overweightness/obesity in comparison to the non-overweight/non-obese group.

### 3.5. Preterm Birth (PB)

**Figure 6:**
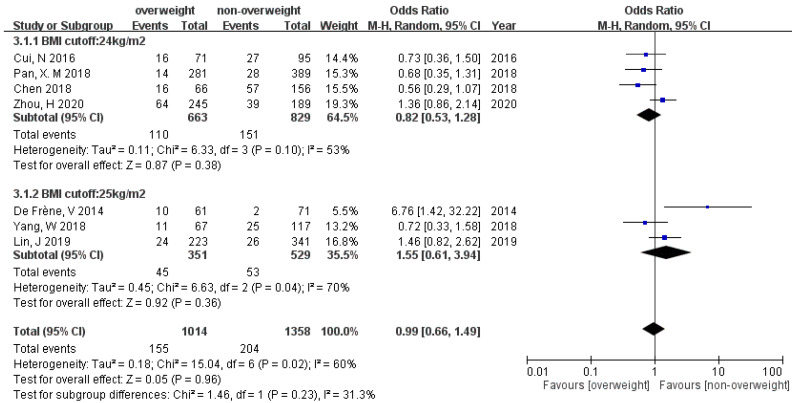
Forest plot of the association between preterm birth and overweightness in women with PCOS.

**Figure 7:**
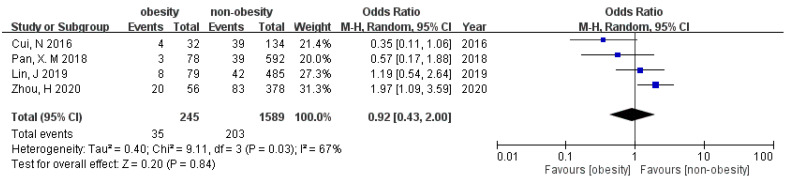
Forest plot of the association between preterm birth and obesity in women with PCOS.

Seven of the studies had analysis for PB. Our meta-analysis did not detect a statistical difference between overweight/obese groups and non-overweight/non-obese groups (OR 0.99 [95% CI 0.66–1.49], and OR 0.92 [95% CI 0.43–2.00]) under random effect model (Figure 6 and Figure 7). A test for publication bias was not performed for the outcomes of preterm birth because the number of eligible studies was small. There is no subgroup effect between BMI cut-off 24 and 25 kg/m^2^ (*p* = 0.23). Because of the limited amount of data in the analysis of the association between preterm birth and obesity, subgroup analysis was not conducted.

## 4. Discussion

In this meta-analysis of cohort studies, we found that overweight or obese women with PCOS had a higher risk of miscarriage (1.71-fold increase in overweight, and 2.00-fold increase in obese) and a lower chance to give a live birth (0.21 reduction rate in overweight and 0.22 reduction rate in obese) compared with those without overweightness or obesity. In this study, the heterogeneity between studies is low. However, regarding to PB, we failed to find a significant difference between groups.

Women with PCOS often present with metabolic dysfunction and IR, which have an impact on body weight and may be potentially related to pregnancy loss [11,41]. A subgroup analysis of meta-analysis concluded that miscarriage in PCOS women was not associated with BMI, but it only analyzed four studies [42]. Another meta-analysis reported women with higher BMI increased risk of spontaneous abortion in PCOS patients undergoing artificial reproductive technologies [43]. Our study enrolling women diagnosed with PCOS, regardless of conception method, also yielded that overweight or obese women had higher odds of miscarriage. Among the included cohorts, Huang et al. and Bailley et al. reported the higher miscarriage rate in the group of women with lower BMI [21,35]. These results were contrary to other studies [12,13,16,17,18,19,20,36,38,39], and may result from the bias from small sample size. There are some possible causes for more miscarriages in overweight/obese women with PCOS. First, obesity is more likely to cause diabetes mellitus because of IR [44,45,46,47,48]. Glucose metabolism is important for endometrial decidualization, and IR may have alteration on endometrial receptivity [49]. Second, gene expression during window of implantation presents differently in obese women with PCOS [50]. Third, it may be related to chronic inflammatory conditions. Oróstica et al. found that similar tumor necrosis factor alpha (TNF-α) serum levels but higher TNF-α signaling with NFκB in endometrium of higher BMI range of PCOS women [51]. Xue et al. further provided a theoretical basis of abnormal endometrium in PCOS women to explain the subfertility of women with PCOS, including anovulation-induced endometrial hyperplasia, hyper-androgenic inhibition of the growth, differentiation and decidualization of the endometrium, IR related disruption of glucose metabolism in the endometrial with subsequent impairment of endometrial receptivity, progesterone resistance of the endometrium and chronic inflammatory change producing a vicious circle that disrupts the physiological endocrine and metabolic microenvironment of the endometrial and affects the receptivity of the endometrium [52]. Furthermore, obesity is associated with impaired ovarian function, poor oocyte quality and decreased reproductive performance by elevated proinflammatory cytokines, such as interleukin 6 (IL-6) and TNFα as well as oxidative stress [53,54].

Live birth rate is significantly higher in non-overweight/non-obese PCOS women with low heterogeneity. One study with subgroup analysis of PCOS women following FET also reported similar result [55]. The decreased live birth rate in overweight PCOS women may result from adverse obstetric outcomes such as pregnancy-induced hypertension, preeclampsia, and GDM, although most of the studies did not report the above outcomes. Overweight or obesity may result in decreased live birth rate due to defective decidualization, causing implantation abnormalities [56]. Abnormal implantation and placentation are associated with pregnancy-induced hypertension, preeclampsia, and IUGR. Besides, a highly significant elevation was recorded in the waist/hip, cholesterol, low-density lipoprotein cholesterol (LDL-C), fasting glucose, LH, LH/FSH ratio, estradiol (E2), and testosterone, while hip circumference, leptin, progesterone, and sex hormone binding globulin (SHBG) were lower in the obese PCOS subjects [57]. One study demonstrated that higher level of triglycerides and lower level of high-density lipoprotein cholesterol (HDL-C) decreased live birth rate owing to impaired oocyte maturation [58].

In regard to the impact of pregestational BMI on PB, previous studies demonstrated inconsistent results in the general population [14,16,20,26,36,38]. One meta-analysis of European, North American, and Australian cohorts reported that both lower and higher maternal pregestational BMI were associated with a higher risk of preterm birth [59], while another meta-analysis conducted in low- and middle-income countries showed that only underweight women had higher risk of preterm birth [60]. As the results remains controversial in the general population, it is not surprising that our study did not have a statistical difference between overweight/obese and non-overweight/non-obese individuals among women with PCOS. In addition, our result was in line with one previous meta-analysis conducted in 2018 [61].

This review adds to the body of literature on the effects of overweight/obesity on the pregnancy outcomes of women with PCOS. There are several strengths to our meta-analysis. First, to our limited knowledge, this meta-analysis directly focused on the association between overweightness/obesity and pregnancy outcomes in women with PCOS, although Bahri et al. had performed meta-analyses comparing pregnancy and neonatal outcomes in women with PCOS and without PCOS [42,61]. The subgroup analyses also reported the effect of BMI on miscarriage and preterm birth in the current study. Nevertheless, the study population in Bahri’s analyses were different from ours, because studies including only PCOS women would not be eligible in meta-analysis conducted by Bahri. In fact, Bahri et al. concluded that miscarriage is not associated with higher BMI value in women with PCOS, and their results could not explain why the live birth rate is lower in PCOS women with higher BMI value. Second, though various criteria for diagnosis of PCOS have been proposed by different societies, including National Institutes of Health (NIH) in 1990, Rotterdam criteria in 2003, and Androgen Excess and PCOS Society (AE-PCOS) in 2006 [62,63,64], most of the cohort studies enrolled into this meta-analysis adopted Rotterdam criteria, and only one study conducted in 1992 using ultrasound image as diagnostic method was included [40]. Third, the definitions of BMI were heterogenous in the literatures [65,66,67,68,69,70,71]. Generally, studies conducted in western countries used WHO classification for nutritional status, whereas studies in China partly used WHO classification, partly used lower cutoff for overweightness and obesity (i.e., BMI 24 kg/m^2^ and 28 kg/m^2^) compared to the WHO classification. The lower cutoff of BMI in China was based on previous studies that Asians generally had lower BMI than Western people [37]. To overcome the concerning that the cut-offs of BMI may potentially influence the results of this study, we added subgroup analysis using different BMI cut-offs.

There are some limitations for this study. First, most of the studies were patients receiving IVF or ICSI treatment. Since patients with PCOS are characterized by chronic anovulation, resulting infertility, it may explain that a lot of studies analyzed individuals with PCOS and infertility treatment. Second, observational studies were included, for which the risk of bias could not be eliminated completely. Third, most of the studies were conducted in China, the results cannot be fully applied in other countries. Finally, we did not discuss other pregnancy outcomes such as GDM, preeclampsia, C/S in our meta-analysis due to scarce information in the studies. Further research aiming on the effect of pre-pregnancy BMI on other pregnancy outcomes in PCOS women is warranted.

## 5. Conclusions

In conclusion, the evidence to date supports a negative association between pre-pregnant overweightness/obesity and live birth rate and a positive association between pre-pregnant overweightness/obesity and miscarriage rate in pregnant women with PCOS, suggesting that the main critical factor contributing to the worse pregnancy outcome may be an early fetal loss in these women with pre-pregnancy overweight/obesity. Therefore, weight control during the preconception period may be warranted and overweight/obese women with PCOS who attempt to get pregnant should be well-informed that reduction of body weight may have a benefit for the subsequent pregnancy outcomes.

## Supplementary Materials

The following supporting information can be downloaded at: https://www.mdpi.com/article/10.3390/ijerph19159094/s1, Table S1: Use MeSH term and Emtree combined with Boolean operators; Table S2: Causes of infertility in the included studies.
