# Supplementary material for: Association between Pre-Pregnancy Overweightness/Obesity and Pregnancy Outcomes in Women with Polycystic Ovary Syndrome: A Systematic Review and Meta-Analysis
Source: Int J Environ Res Public Health. 2022 Jul 26;19(15):9094. doi: 10.3390/ijerph19159094 (PMC9332574; doi:10.3390/ijerph19159094)
Supplement: Supplementary file 1 [file ijerph-19-09094-s001.zip › ijerph-1812324-supplementary.pdf]

**Table S1.** Use MeSH term and Emtree combined with Boolean operators.

| **PubMed** |
|---|
| (Polycystic ovary syndrome) AND (Obesity, Maternal OR Obesity in Pregnancy OR Overweight OR Body Mass Index) AND (Miscarriage OR Spontaneous Abortion OR Fetal Demise OR Fetal Deaths OR Pregnancy-Induced Diabetes OR Gestational Diabetes Mellitus OR Pregnancy-induced hypertension OR Gestational Hypertension OR Pregnancy Transient Hypertension OR Preeclampsia OR Eclampsia OR Cesarean Sections OR Preterm Birth OR Premature Births OR Fetal Macrosomias OR Intrauterine Growth Restriction OR Fetal Growth Restriction) |
| **EMBASE** |
| (Polycystic ovary syndrome) AND (Maternal obesity OR body mass) AND (Spontaneous Abortion OR Fetal death OR Pregnancy diabetes mellitus OR Maternal hypertension OR Preeclampsia OR Eclampsia OR Cesarean Section OR Immature and premature labor OR Macrosomia OR Intrauterine Growth Retardation) |
| **Web of science** |
| (Polycystic ovary syndrome) AND (Maternal obesity OR body mass) AND (Spontaneous Abortion OR Fetal death OR Pregnancy diabetes mellitus OR Maternal hypertension OR Preeclampsia OR Eclampsia OR Cesarean Section OR Immature and premature labor OR Macrosomia OR Intrauterine Growth Retardation) |
| **Cochrane database** |
| (Polycystic ovary syndrome) AND (Maternal obesity OR body mass) AND (Spontaneous Abortion OR Fetal death OR Pregnancy diabetes mellitus OR Maternal hypertension OR Preeclampsia OR Eclampsia OR Cesarean Section OR Immature and premature labor OR Macrosomia OR Intrauterine Growth Retardation) |

**Table S2.** Egger's test of outcomes of miscarriage and live birth in sensitivity analyses.

| **Miscarriage** | | **Live birth** | |
|---|---|---|---|
| Sensitivity analysis 1 | p= 0.14 | Sensitivity analysis 1 | p= 0.36 |
| Sensitivity analysis 2 | p= 0.47 | Sensitivity analysis 2 | p= 0.08 |
| Sensitivity analysis 3 | p= 0.88 | Sensitivity analysis 3 | p= 0.22 |
